# 

**Published:** 1881-12-20

**Authors:** 


### TABLE OF CONTENTS.

ORIGINAL AND SELECTED ARTICLES.

Clinical Lectnre—Asthma, Vesicular Emphysema, Hay Fever, by Robert C. Word, M. D., Professor of Physiology and Lecturer on Hygiene in the Southern Medical College, Atlanta, Ga., 441. Viburnum Prunifolium, by A. G. Smythe, M. D., of Mississippi, 449. Cancrum Oris, by B. F. Duke, M. D., of Mississippi, 451. The Value of Hydrocyanate of Iron in the Treatment of Neuralgia, by J. T. Stovall, M. D., of Columbia, Ala., 452. Some Uses of Chloral Hydrate, by G. A. Collamore, M. D., of Toledo, Ohio, 454 Transmission of Tuberculosis by Vaccination; False Report, 457,

ABSTRACTS AND GLEANINGS.

Belladonna as an Antidote to Opium Poisoning, 458. Prolapse of the Ovaries, 459. A Successful Operation of Gastrotomy for Intussusception, 460. The Best Position for Women in Labor, 461. Sodium Phosphate in Habitual Colic, 462. The Limit of Danger in the use of Anæsthetics; Small-Pox in Richmond, 463. Restoring the Heart's Action when it has Ceased to Beat, 464. Ipecacuanha in Jaundice; Pneumonia Aborted with Quinine, 465. Catching Cold, 466. New Treatment for Syphilis; Treatment of Chronic Ulcers of the Leg, 467. Treatment of Hernias of Long Standing; Coca a Cure for Morphineism, 468. Alcohol; Dietetic Treatment of Intestinal Fluxes; Capsulotomy, 469. Mr. Jefferson Davis; New Test of Albumen in Urine; Comparative Proportion of Physicians to Inhabitants in Different Countries; Hot Rectal Douche, 470.

SCIENTIFIC ITEMS.

Grafting the Tomato to the Potato; Meteorites; A Movement, etc., 471. Snakes; Quicksilver; Cold Fire; Stain for Mahogany Cherry, 472.

PRACTICAL NOTES AND FORMULÆ.

Diarrhœal Disorders of Infancy; Aquarium Cement; English Hop Bitters; Spasmodic Dysmenorrhœa, 473. For Worms; Inflamed Hemorrhoids; Styptic Colloid; Repression of Heat Production; Ointment for Scabies; Ointment, 474. For Habitual Constipation; Headache, 475. Case of Gonorrhœa; Treatment of Hydrocele, 476.

EDITORIALS AND MISCELLANEOUS.

Our Journal for 1882; Alienist and Neurologist; Practical Journalism, 477. Pharmaceutical Board in Georgia; Dr. J. Miller Fothergill on the use of Maltine; Lady Pharm cists, 478. Book Notices; Receipts, 479. Special Notices, 480.

